# Data management plans as linked open data: exploiting ARGOS FAIR and machine actionable outputs in the OpenAIRE research graph

**DOI:** 10.1186/s13326-023-00297-5

**Published:** 2023-11-02

**Authors:** Elli Papadopoulou, Alessia Bardi, George Kakaletris, Diamadis Tziotzios, Paolo Manghi, Natalia Manola

**Affiliations:** 1 OpenAIRE AMKE, 6 Artemidos, Athens, 15125 Greece; 2 Consiglio Nazionale delle Ricerche, 1 Via Moruzzi 56124, Pisa, Italy; 3 Communication and Information Technologies Experts S.A, 22 Omiriou, Athens, 16122 Greece

**Keywords:** Research Data Management, Data Management Plans, FAIR DMPs, Machine Actionable, Research Graphs, Knowledge graphs, Open Science

## Abstract

**Background:**

Open Science Graphs (OSGs) are scientific knowledge graphs representing different entities of the research lifecycle (e.g. projects, people, research outcomes, institutions) and the relationships among them. They present a contextualized view of current research that supports discovery, re-use, reproducibility, monitoring, transparency and omni-comprehensive assessment. A Data Management Plan (DMP) contains information concerning both the research processes and the data collected, generated and/or re-used during a project’s lifetime. Automated solutions and workflows that connect DMPs with the actual data and other contextual information (e.g., publications, fundings) are missing from the landscape. DMPs being submitted as deliverables also limit their findability. In an open and FAIR-enabling research ecosystem information linking between research processes and research outputs is essential. ARGOS tool for FAIR data management contributes to the OpenAIRE Research Graph (RG) and utilises its underlying services and trusted sources to progressively automate validation and automations of Research Data Management (RDM) practices.

**Results:**

A comparative analysis was conducted between the data models of ARGOS and OpenAIRE Research Graph against the DMP Common Standard. Following this, we extended ARGOS with export format converters and semantic tagging, and the OpenAIRE RG with a DMP entity and semantics between existing entities and relationships. This enabled the integration of ARGOS machine actionable DMPs (ma-DMPs) to the OpenAIRE OSG, enriching and exposing DMPs as FAIR outputs.

**Conclusions:**

This paper, to our knowledge, is the first to introduce exposing ma-DMPs in OSGs and making the link between OSGs and DMPs, introducing the latter as entities in the research lifecycle. Further, it provides insight to ARGOS DMP service interoperability practices and integrations to populate the OpenAIRE Research Graph with DMP entities and relationships and strengthen both FAIRness of outputs as well as information exchange in a standard way.

## Background

Funders’ and institutions’ mandates on open and FAIR [7]^1^ data management resulted in new demands for responsible research conduct, affecting traditional practices followed by stakeholders and posing a dynamic cultural shift in science and scholarly communication, globally. The UNESCO Recommendation on Open Science is a clear indication towards that direction, uniting nations and their leaders into acknowledging the benefits and acting upon Open Science adoption for sustainable and prosperous societies [17]. In Europe, responsible data management is considered a vital part of Open Science incorporated in Framework Programmes (Horizon2020, HorizonEurope), policy documents (Recommendation on access to and preservation of scientific information) [13] and implementation actions (European Open Science Cloud) [2]. Researchers and students are expected to embrace and thrive in the new paradigm of Open Science since their grants or graduations heavily rely on complying with Research Data Management (RDM) policies. In this context, one way RDM can be supported is through the use of tools that facilitate the writing of Data Management Plans (DMPs).

DMPs have traditionally been *free-form text documents* that describe the processes, tools, and effort undertaken in data handling and management activities, including FAIR implementation, throughout a project lifecycle. The structure and expected content of the DMPs is provided in *model templates* prepared by the respective authorities who will receive researchers’ DMPs upon its submission. The most common requirements for DMPs are: (1) Data description and collection or re-use of existing data, (2) Documentation and data quality, (3) Storage and backup during the research process, (4) Legal and ethical requirements, codes of conduct, (5) Data sharing and long-term preservation, (6) Data management responsibilities and resources [20]. Today, DMPs are expanding their purpose to include management of software and other research outputs [21].

There have been early efforts to facilitate the DMP authoring process involving the development of software that guides researchers into the DMP contents and instructs them on how to answer the required questions to then download and share their work as .pdf files. DMPRoadmap^2^ is the first software for DMPs, developed back in 2010-11 by the Digital Curation Centre and the University of California Curation Center. As the first tool to provide DMP workflows and guidance, DMPRoadmap had been widely adopted by the scientific community. In the later years, however, community needs have raised to accommodate intrinsic elements of the evolving FAIR ecosystems (e.g. Persistent Identifiers - PIDs, standards, machine readable licenses) calling for new research data services to be built and others to be reformed according to those standards [18]. Following such demand, new DMP software solutions are developed, such as ARGOS^3^, Data Stewardship Wizard^4^ and the Research Data Management Organizer^5^.

Recently, Research and Innovation (R&I) ecosystems have started to realize Knowledge Graphs for their ability to present a contextualized view of current research that supports discovery, re-use, reproducibility, monitoring, transparency and omni-comprehensive assessment [1]. In particular, Open Science Graphs (OSGs) are scientific knowledge graphs representing different entities of the research lifecycle (e.g. projects, people, research outcomes, institutions) and the relationships among them. They provide insight on the wealth of information in Open Science environments which, according to RDA^6^, can be classified to at least the following subsets: thematic graphs, citation graphs and monitoring graphs^7^. Examples of OSGs are Open Citations^8^, which focuses on bibliographic and citation data; Research Graph^9^, which connects scientific literature, research data, project grants and researchers; the Open Research Knowledge Graph, which focuses on research papers and the knowledge available in their full-texts; the FREYA PID Graph^10^, which includes interlinked entities provided they are assigned a persistent identifier; and the OpenAIRE Research Graph (OpenAIRE RG)^11^[5], which includes descriptive metadata, provenance metadata and links between research results of any kind (literature, software, datasets, and other), organisations, grants, research communities and infrastructures.

Although important, DMPs are not yet integral parts of the ecosystem, and consequently, OSGs. Moreover, the adoption of ma-DMPs, i.e. machine actionable exports of DMPs that provide a structured way of organizing their content, is limited. Until today, DMPs are typically produced and submitted as deliverables outside of scientific workflows that support the publication of FAIR research outputs. Those practices pose several limitations on the DMP lifecycle, from the discovery and re-use of DMPs, to their enhancement with other information and further exploitation [15, 16].

In this respect, ARGOS (argos.openaire.eu) [6] was built and integrated into OpenAIRE to provide to the scientific community a solution that produces and publishes FAIR and ‘*machine actionable*’ DMPs (ma-DMPs) [12]. OpenAIRE is the largest source for Open Access Scholarly Communication in Europe and a pillar infrastructure to the architecture and operations of the EOSC^12^. Since 2007, OpenAIRE has enabled interoperability of literature and data repositories [9, 10] and has aggregated, deduplicated, contextualized and made available metadata from millions of scientific information building the OpenAIRE RG. In order to enhance DMP practices and FAIRness, ARGOS explored the possibilities offered by other underlying services of the OpenAIRE service portfolio.

First, ARGOS applied the DMP Commons Standard [18] to achieve a minimum layer of interoperability to its DMP outputs. Then, ARGOS moved with publishing its DMPs to Zenodo to complete the DMP *lifecycle* and *enhance* FAIRification of its outputs (DMPs are assigned DOIs and licenses). Finally, with DMPs being exposed in OpenAIRE (via Zenodo), the OpenAIRE RG became ARGOS main trusted source to: populate DMPs with standardized information, and create links with other outputs and activities (datasets, projects, funders).

This paper, to our knowledge, is the first to explore the modelling, representation and linking of DMPs in OSGs. It explains the mapping activity that led to ARGOS DMP service integration with the OpenAIRE RG. The paper presents ARGOS as a case study for FAIR and ma-DMPs exposed in OSGs and highlights the new potential unlocked in the DMP landscape, from discovery to enrichment and exploitation.

## Construction and content

ARGOS DMP service is based on the OpenDMP open-source software^13^ that was developed in collaboration between OpenAIRE and EUDAT CDI^14^. OpenDMP offers the possibility of semantic tagging in a multi-granular manner (dataset level) and of connecting with other services and workflows to validate and exchange information, automate the DMP publication and writing processes and apply the FAIR principles to its outputs.

ARGOS is an instance of the OpenDMP open source software^15^, which is configured in the OpenAIRE ecosystem, and is available through the OpenAIRE Service catalogue^16^ and the European Open Science Cloud (EOSC)^17^. In this section, we present how the OpenDMP ARGOS instance builds on and produces FAIR and machine actionable processes and outputs. Those are seen as prerequisites to the mapping activity between ARGOS and the OpenAIRE RG because they enrich the DMP content and help produce better mapping results.

### A. OpenDMP model and interoperability

The OpenDMP software features, among other things, a Template editor which is used by Administrators who define personalised structures and rules (e.g. mandatory fields, types of input supported, such as APIs) for DMPs according to RDM policies. An example is the H2020 DMPs template [11] that can be used in ARGOS to create DMPs for projects funded under the European Commission H2020 framework programmes [4]^18^.

The Template editor is a key enabler of compatibility with the RDA standard as it incorporates a mechanism that maps ARGOS OpenDMP fields contained in templates to template entities, including RDA dataset entities.

OpenDMP has two more editors: the DMP Editor and the Dataset editor (embedded in the DMP editor) which are used by researchers who describe their datasets/outputs (e.g. how FAIR principles were followed throughout the RDM lifecycle) and project information (e.g. funding and beneficiaries) in DMPs. DMPs produced in OpenDMP enable values in DMPs to be fetched from external APIs, like OpenAIRE APIs^19^,ORCID API^20^, and EOSC API^21^.

An essential element for achieving interoperability with standards and 3rd party systems, given the flexibility of the ARGOS underlying data model, which assumes almost unlimited user-driven structuring of dataset descriptions, is the concept of “semantic tags”. Semantic tags are tags that are attached to elements of a data set description template by their authors, so that, in due time, they utilized properly by an informed component that handles an intended action, be it an export or an interaction with an external system.

### B. ARGOS lifecycle and FAIR DMPs

A DMP’s lifecycle in ARGOS consists of a predefined number of internal states followed by versioning and publication workflows. A DMP is in draft state while being composed, and while draft it might be either validated (i.e. passing all the requirements of the dataset templates that each of its contained datasets conform to) or non-validated, which is computed substate. Validated DMPs can advance to a finalized state, where editing is restricted, unless the DMP is reverted to draft again. The DMP may be published both internally, i.e. listing of published DMPs offered by the service, or externally.

**Fig. 1:**
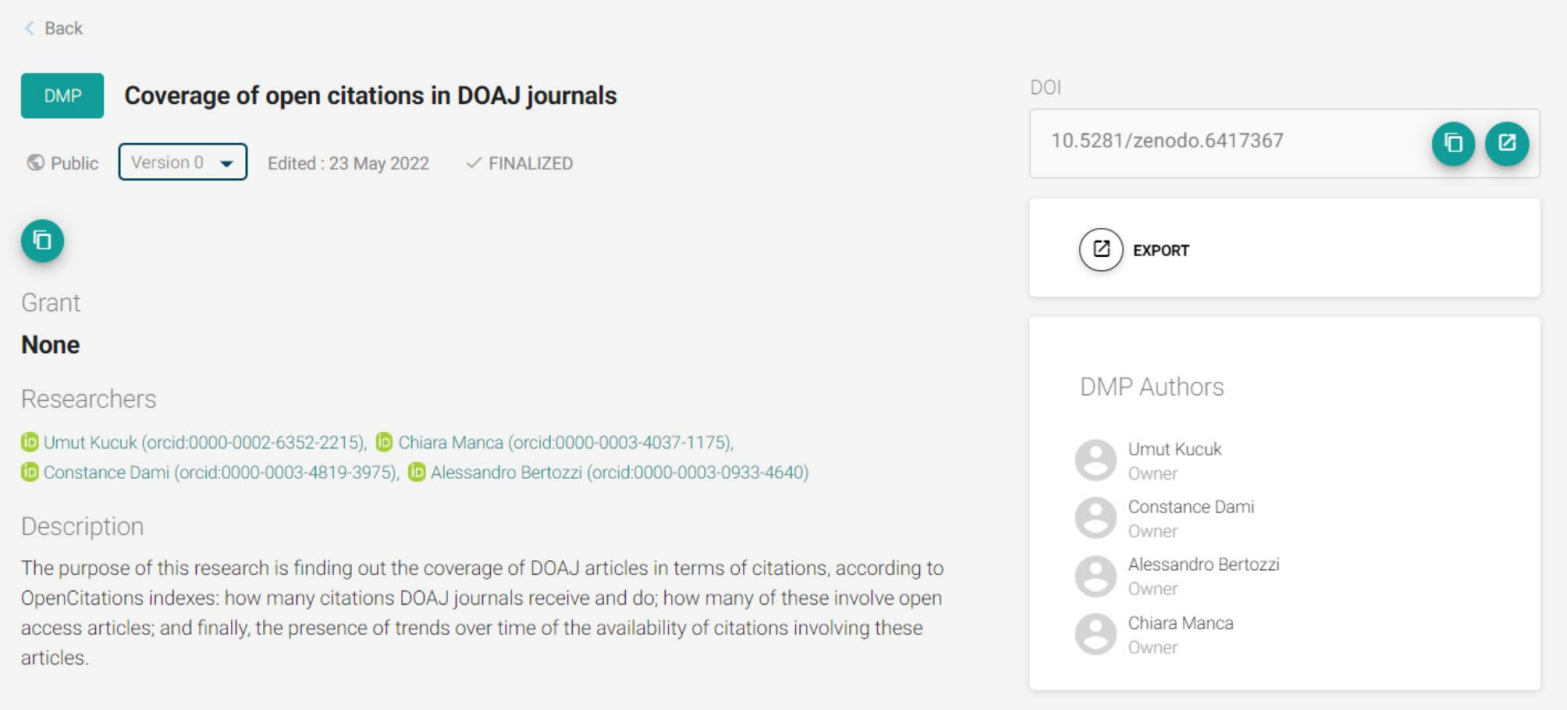
Published DMP record (with DOI) in ARGOS platform

**Fig. 2:**
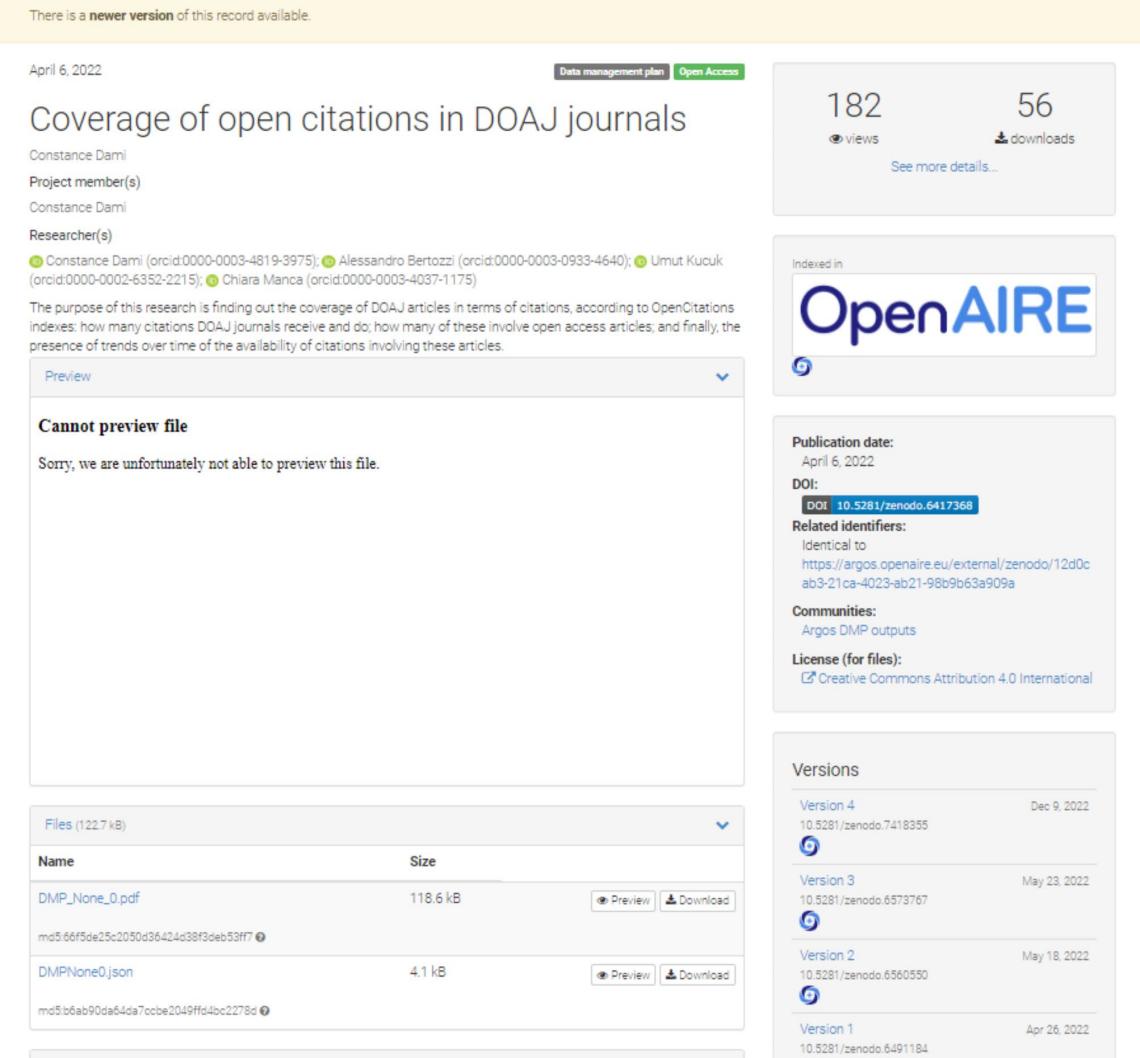
The published DMP of Fig. 1 in Zenodo exposed in both .pdf and .json

As shown in Figs. 1 and 2, ARGOS is fully integrated with Zenodo^22^, the catch-all repository of OpenAIRE hosted by CERN, and thus offers the option to publish DMPs as outputs in an open and FAIR manner (.pdf and .json DMP representations), by assigning DOIs^23^, descriptive metadata, and licenses and by supporting DMPs as living documents through versioning.

Once a DOI is issued from Zenodo, reverting a DMP to draft state is not possible and a new version must be created in order to add modifications. Following one cycle of publication, new versions of a DMP may be created and linked to their predecessor ones, while the latter continue to survive in the system for reference.

**Fig. 3:**
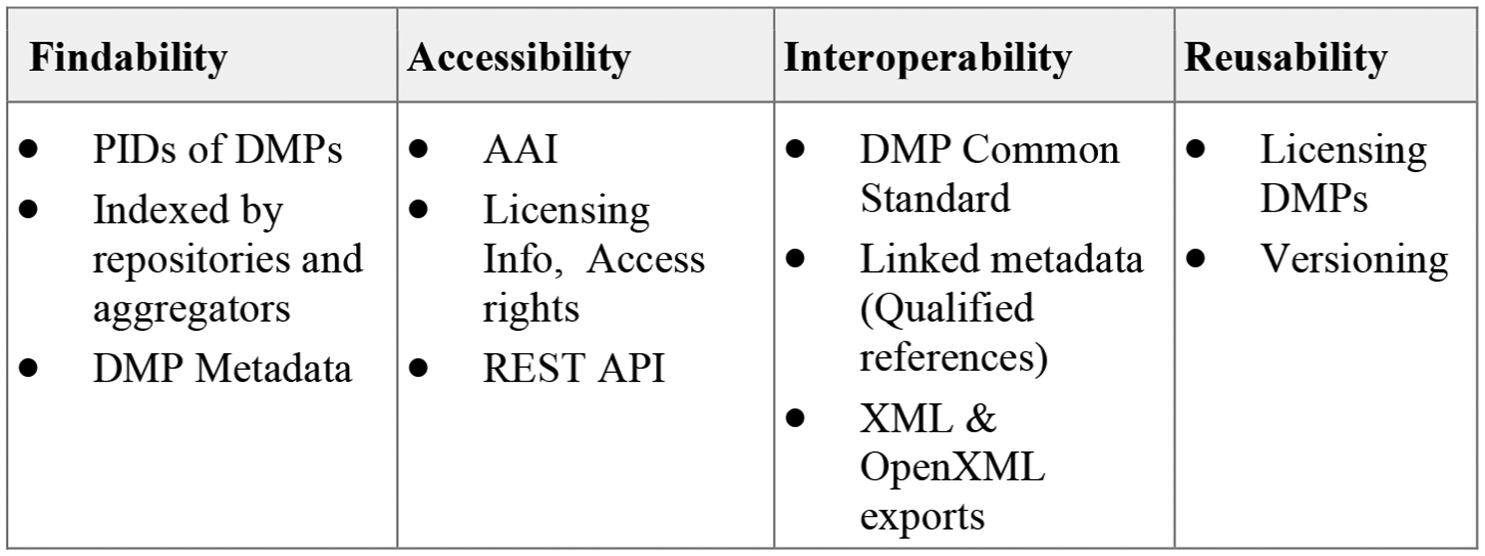
FAIR DMPs in ARGOS

The following table highlights how the ARGOS OpenDMP instance has enabled FAIRification of its outputs Fig. 3.

In summary, ARGOS DMPs are findable because they are minted DOIs directly from Zenodo, which in turn is registered in the re3data data repositories registry^24^ and is indexed by OpenAIRE. Metadata of ARGOS DMPs include PIDs, such as ORCIDs for researchers, and are exposed to Zenodo, shared under CC0 license. To access work performed on DMPs, OpenDMP supports a number of configurable providers for login to the system (SAML 2.0/OpenID Connect/ OAuth2.0), including OpenAIRE for the ARGOS instance. Additionally, ARGOS DMPs are provided with access rights (open and restricted) to allow specific conditions to be set along with their license information. To make DMPs interoperable and enable their exchange, apart from the DMP Common Standard supported, DMPs are exported in formats that are machine readable and machine actionable, namely .xml and .json. The xml format is a full native representation of a DMP, exposing ARGOS model, containing elements accompanied with tags that may be utilized for interoperability with other systems. The .json format conforms to the specs introduced by RDA ma-DMP working group. From the Research Graph, links are created between ARGOS DMPs and other outputs. Furthermore, DMPs can be versioned at any time of the DMP lifecycle and are assigned licenses to enhance reusability practices.

## Methods

The process of integrating ARGOS FAIR ma-DMPs with the OpenAIRE Research Graph concentrated on mapping the data models of OpenAIRE [5], OpenDMP [19] and the DMP Common Standard. This was a vital step to build the framework for information exchange and semantics adoption between a ma-DMP service and an Open Science Graph.

The aim was to identify what information captured in DMPs was already available in the graph, and vice versa, as well as how entities and properties are expressed in all settings, thus contributing to the mapping results. Since the DMP Common Standard is used to exchange DMPs in a standard way, it had a prominent place in this activity for providing the minimum set of DMP related properties that comprise a ma-DMP. Then, identified gaps and weaknesses were reported and prioritized for implementation as new entities and properties in both ARGOS and the OpenAIRE RG.

The mappings were further analysed to report possible new relationships and properties that were not included in metadata records of DMPs found in OpenAIRE, but that are instead available in ma-DMPs like those produced and exposed by ARGOS.

Of particular interest is the RDA Hackathon^25^ that was organized in May 2020, which provided the opportunity for active conversation among DMP providers and the RDA community^26^. ARGOS winning the Hackathon accelerated advancements in terms of interoperability and machine-actionability, also with respect to integrations with the OpenAIRE RG.

## Results

The comparative study [14] between OpenDMP, OpenAIRE RG and the DMP Common standard models revealed common entities, properties, and relationships, clearly portraying the state-of-the-art for DMPs as well as the opportunities arising with the adoption and publication of ma-DMPs in OSGs.

### Mapping between ARGOS and the DMP Common Standard

**Fig. 4:**
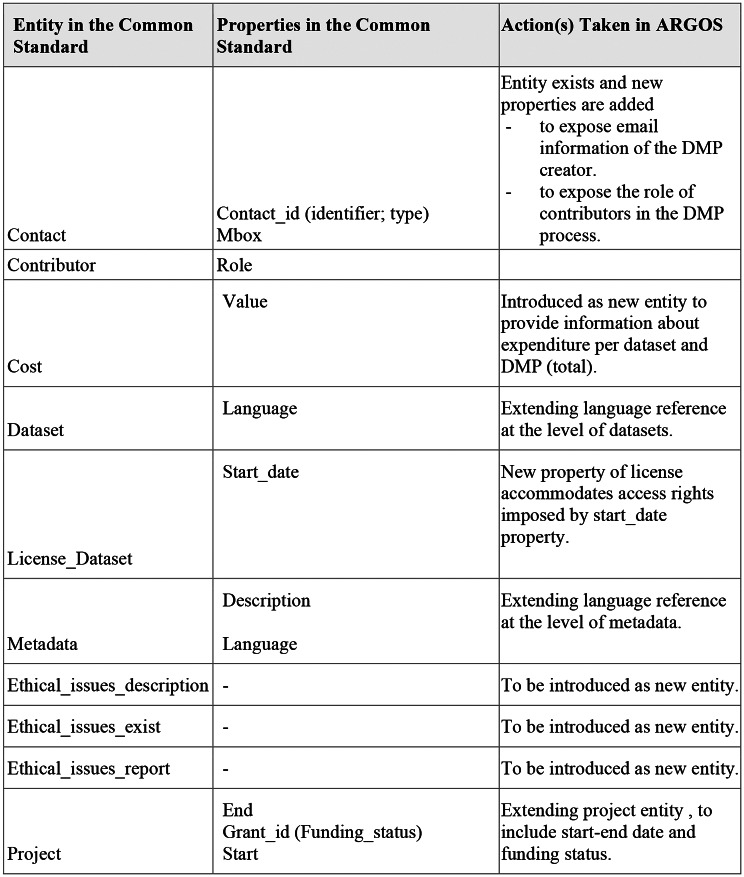
DMP Common Standard elements not in ARGOS

The mapping highlighted the need for updates in ARGOS to be fully compliant to the RDA standard and strengthen its ma-DMP outputs. In Fig. 4, we show the entities and their specific properties that were found to be absent from ARGOS.

### Mapping between the OpenAIRE RG and DMP Common Standard

Given that some researchers publish their DMPs in repositories, the OpenAIRE RG already included metadata about few DMPs made available by content providers as records compliant to the OpenAIRE guidelines [9, 10]. Metadata includes properties and relationships that are useful for findability (persistent identifiers), accessibility (landing pages and download URLs of different versions of DMPs, access right information), citation and discovery (bibliographic metadata properties), and for tracking (links to funding grants and involved organisations). Observing the DMPs that were available in the OpenAIRE RG, indicated that they were “disguised as project deliverables”. Hence, OpenAIRE exploited the COAR vocabulary which introduced resource type “DMP”^27^ in the latest release to enable the proper typing of DMPs that would clearly identify them on the OpenAIRE RG. Following that, Zenodo introduced a new type of resource so that users can select the type “Data Management Plan” upon deposition. Because ARGOS uses Zenodo as its publishing mechanism and Zenodo is exploited by OpenAIRE as one of its core data sources, ARGOS published ma-DMPs become immediately an integral part of the OpenAIRE RG. That way, ARGOS ma-DMPs are exposed with proper resource_type and are treated as independent entities in the OpenAIRE RG contributing to making searching for DMPs and their links with other outputs an established process.

**Fig. 5:**
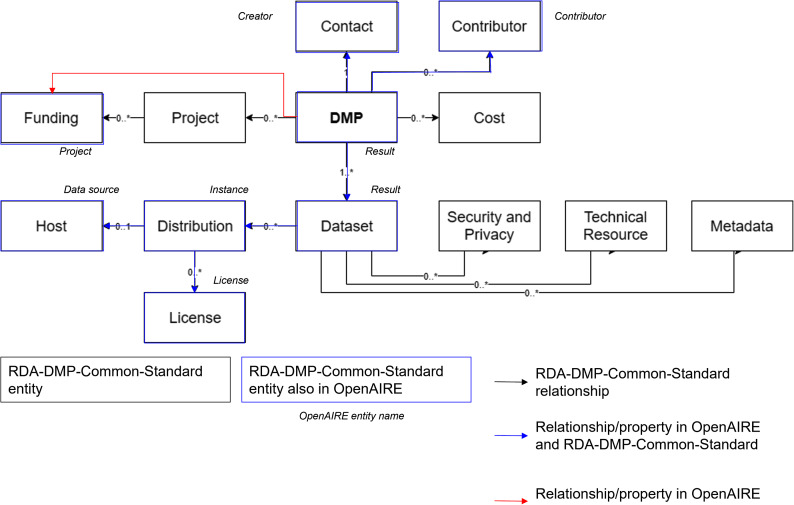
RDA standard and the OpenAIRE Research Graph Model

As it is shown in Fig. 5, when analysing the mapping of the OpenAIRE and DMP Common Standard models, we observed that most of the ma-DMP entities can be mapped directly to the OpenAIRE RG Entities.

Those entities are:

- Identifier (*DMP_id*), indicating the persistent identifier of the DMP record.
- Title of the DMP.
- Date of creation and modification of the DMP record.
- Description regarding the context that the DMP is created.

**Fig. 6:**
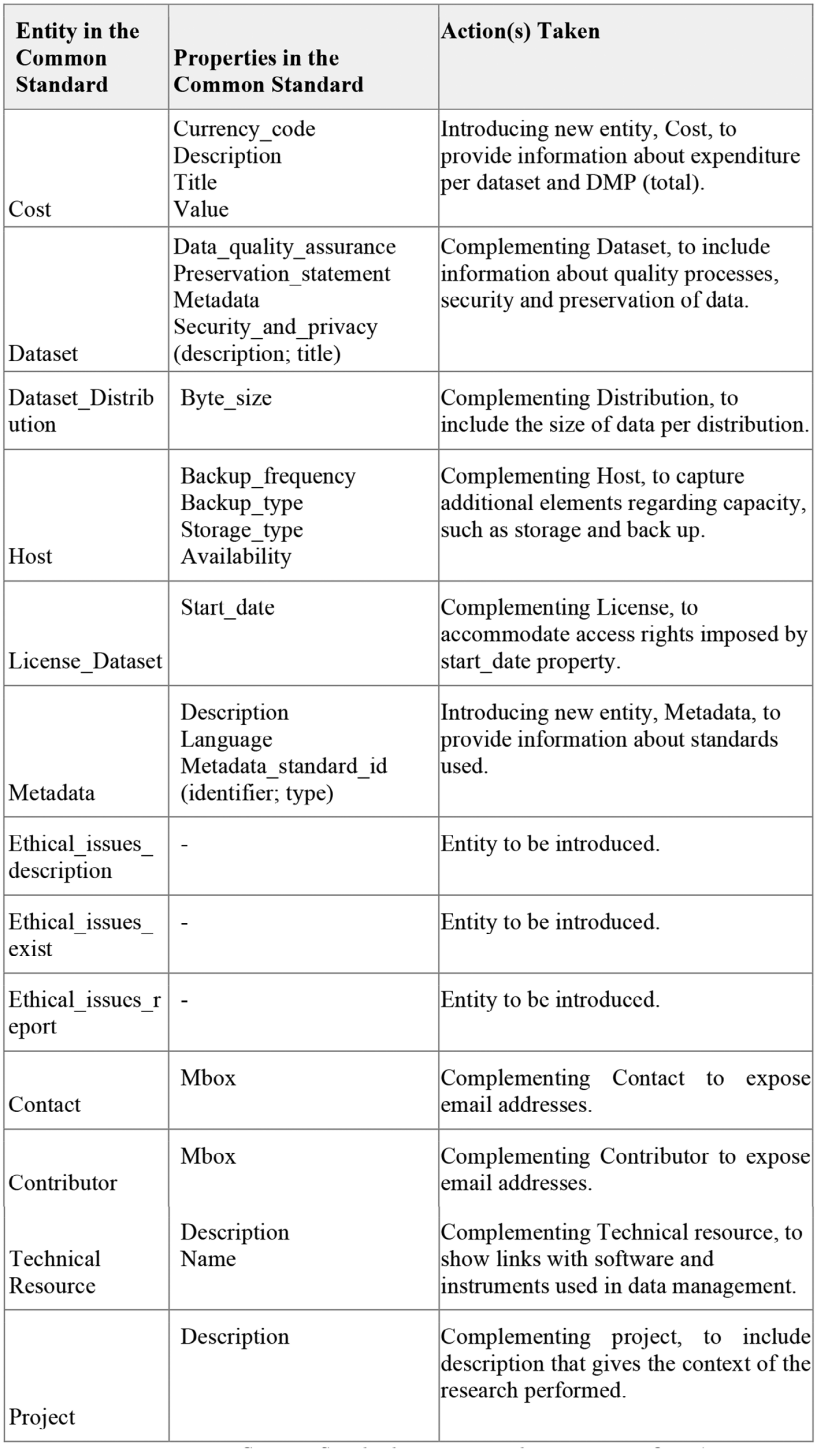
DMP Common Standard properties and entities not in OpenAIRE

Additionally, Fig. 6 shows that the mapping activity identified areas where the OpenAIRE RG can be strengthened.

**Fig. 7:**
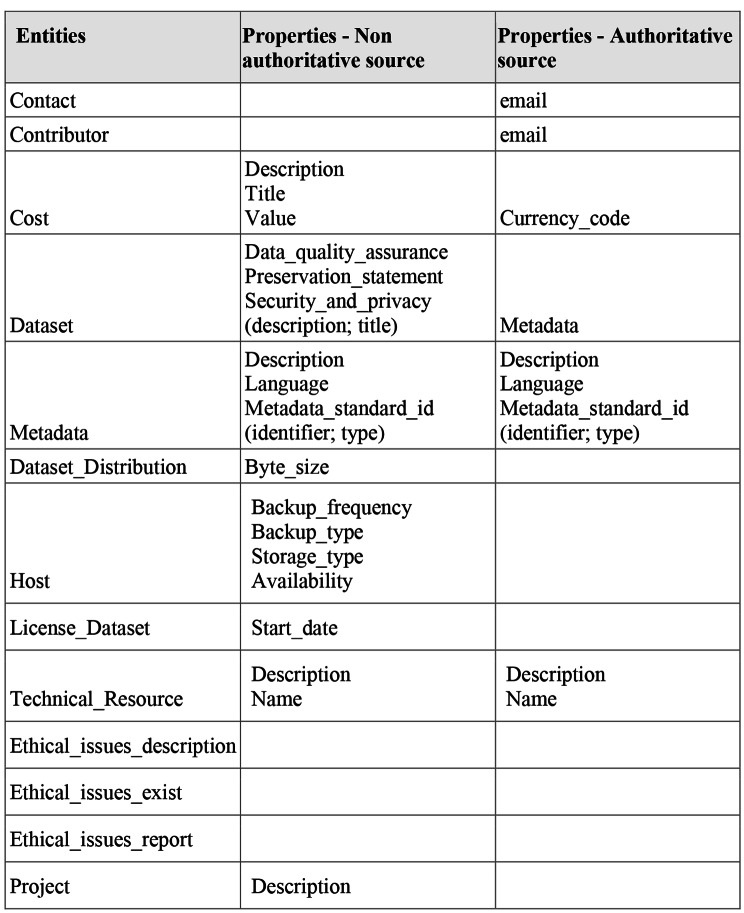
ARGOs DMP elements enriching the OpenAIRE RG

ARGOS ma-DMPs enrich the OpenAIRE RG with the identified entities and properties using researchers’ input. This input might be inferred from an API selection, such as. OpenAIRE, EOSC, Zenodo, (authoritative source) or be the compilation of free text statements provided in the DMP (non authoritative source). In Fig. 7, we show which entities and properties and in what way ARGOS can provide them to enrich the graph with missing information.

Particularly for dataset metadata about ethics, security, quality, and preservation available in ma-DMPs, OpenAIRE could consider revisiting its guidelines [9, 10] so that this information always reaches the OpenAIRE data model from data deposits in OpenAIRE compliant repositories.

Besides expanding information captured in the OpenAIRE RG, additional efforts are needed to create value out of this captured information. Below we describe what relation types are available in the OpenAIRE RG and what changed to accommodate links between ma-DMPs and other entities.

The OpenAIRE Research Graph features a dedicated relation type between research products and project grants: “isProducedBy/produces” (a product is produced by a project; a project produces research product). This type of relationship can exist between projects and research products of any type (publications, datasets, software and other types of research products), and is used by OpenAIRE to model the association between DMPs and project grants. Considering the importance assigned by funders to DMPs (e.g. for H2020 grants, the DMPs is a “living deliverable” that must be updated frequently during the lifetime of the project), and to support the development of added-value services on top of machine actionable DMPs, the OpenAIRE Research Graph will include a relationship with a dedicated semantics “hasDMP”/”hasProject”.

Interestingly, the DMPs that are currently available in the OpenAIRE Research Graph do not include explicit links to the datasets they refer to. Clearly, such links may not exist when the first version of a DMP is published - because the datasets may not yet exist -, but the expectation is that the datasets are made available during the life-time of the project. Thanks to the different versions of the DMPs, therefore, it would be possible for OpenAIRE to add relationships with specific semantics between a DMP and the referred datasets. The relationships available in the OpenAIRE Research Graph, inspired from CERIF [8] and Datacite^28^, do not include a specific semantics that could depict the association between a DMP and its datasets. OpenAIRE is therefore planning to add a new relationship with semantics “hasDataset”, drawn from the core ontology of the RDA standard [3], and a corresponding inverse relationship, which is instead not defined in the standard, to link datasets to the DMPs (“hasDMP”).

### Utility and discussion

Observations made during the mapping activity of entities and properties between the three data models of OpenDMP, OpenAIRE RG and DMP Common Standard showed that when direct mapping couldn’t be fulfilled, information might still be able to be found in more abstract/ general fields of OpenAIRE or ARGOS though diverged in cardinality and / or data type; some information might still be covered by ARGOS DMP outputs as they enter the OpenAIRE RG or tweaked to accommodate the needs of ma-DMPs documentation or, rarely, they may be omitted in information exchange. OpenAIRE, also, highlighted the value added in contextualising ma-DMPs as they contain structured and specialised information, especially about datasets, which cannot be found in original / traditional DMP documents.

Searching OpenAIRE for DMP outputs outside ARGOS, showed that DMPs are still typed as generic publications or reports, and not as data management plans. The introduction of a specific term for DMPs in global vocabularies, such as COAR’s, is expected to improve the current status, although the adoption of the new term may take some time to be widespread. The recent update of the Datacite metadata schema^29^ which includes DMP IDs, will significantly contribute towards wider adoption by service providers.

Furthermore, in order to conform to the needs of ma-DMP fixed schema, without losing the versatility of its templating mechanism, ARGOS software follows an approach that engages an extensible mechanism for attaching export format converters (ma-DMP being one of them) and semantic tagging of template elements that can be used “at-will” by those converters. The ma-DMP converter makes use of its knowledge of the fixed part of ARGOS data model as well as attributes attached to various dataset description fields in order to pick the data required for a ma-DMP file.

The flexibility of ARGOS machine actionable templating system combined with the integration of the publication mechanism of Zenodo and the interlinking with the OpenAIRE RG is crucial as it fosters change in the scene for DMPs content, distribution, exploitation, and reusability. According to the recent study of DMPs in Horizon 2020 [15], there are more than 1500 publicly available DMPs in CORDIS that most of them do not indicate the ways in which they can be re-distributed and used. That practice reverts the situation to the known problem of open access. The licenses assigned to ARGOS DMPs and the access rights indicated in its context, eliminate such confusion. Moreover, another study on Monitoring the Open Access Policy of Horizon 2020 [16], proves the importance of individual dataset descriptions in the exploitation of datasets and DMPs. That way, all datasets described in DMPs become individually identifiable in a granular way that links them with their associated metadata, repositories, back up policies etc. Similarly, re-used datasets become identifiable and can be further quantified, analysed and validated in reproducibility studies.

Moreover, it is foreseen that the OpenAIRE data model will be complemented with information that is currently not present, such as DMP cost or metadata. Cost information is not crucial to improve the level of FAIRness of DMPs or datasets, but it might enable analysis about data management costs useful to funders, organisations, and project administrators. Similarly, this kind of information is potentially useful to studies about responsible research and may be integrated to serve specific analysis or use cases related to RRI (Responsible Research & Innovation) monitoring.

Finally, this case study explored information carried out in published, i.e. DOIed DMPs. An opportunity that ARGOS has already started to exploit with Zenodo is pre-filling of ma-DMPs with information from deposited metadata records. This automation highly contributes to the needed cultural shift that Open Science cultivates and incentivizes researchers to follow best practices while minimizing their compliance effort. Among the issues being examined in this implementation are capturing of the state of datasets that are pre-filled (e.g. reused dataset in a DMP) and adopting the pre-filling mechanism in other repository software, such as Dataverse. Further connections with data analysis PIDs are expected in the context of the Research Analysis Identifier System - RAISE project.

## Conclusion

The paper aimed to provide a case study of how machine actionable DMPs can be contextualised and exploited in an Open Science Graph, and specifically the OpenAIRE Research Graph. ARGOS, the OpenAIRE DMP service, contributed to this work by paving the way to exposing ma-DMPs in OpenAIRE.

The paper explained how ARGOS is structured, how it applied the RDA standard to increase interoperability and machine-actionability of its outputs, how it integrated with other services to increase openness and FAIRness, and how it enriched the OpenAIRE RG. This is an ongoing effort between ARGOS and the OpenAIRE RG, supported thanks to the OpenAIRE API providing information about organizations, data sources and datasets, obtained by collecting metadata records from more than 12 K trusted scholarly communication sources (including Datacite, Crossref, re3data, OpenDOAR, Grid.ac).

The results of the comparative analysis that was performed between the data models of ARGOS and OpenAIRE Research Graph against the DMP Common Standard extended ARGOS with export format converters and semantic tagging, and the OpenAIRE RG with a DMP entity and semantics between existing entities and relationships. This enabled the integration of ARGOS machine actionable DMPs (ma-DMPs) to the OpenAIRE OSG, enriching and exposing DMPs as FAIR outputs.

Today, the work of this paper has shaped ARGOS in becoming the only DMP provider that completes the lifecycle by exposing its DMPs, including the ma-DMPs, directly to repositories and the only one to apply the whole set of the FAIR principles to its outputs so that DMPs are Findable, Accessible, Interoperable and Reusable.

## Data Availability

The datasets generated and/or analysed during the current study are available in the ZENODO repository, 10.5281/zenodo.5047766.

## Footnotes

1 Findable, Accessible, Interoperable and Reusable.

2 DMPRoadmap source code: https://github.com/DMPRoadmap/roadmap.

3 ARGOS/OpenDMP source code: https://code-repo.d4science.org/MaDgiK-CITE/ARGOS.

4 Data Stewardship source code: https://github.com/ds-wizard/.

5 RDMO source code: https://github.com/rdmorganiser.

6 Research Data Alliance.

7 Open Science Graphs for FAIR data Interest Group: https://www.rd-alliance.org/groups/open-science-graphs-fair-data-ig.

8 https://opencitations.net/.

9 https://researchgraph.org/.

10 https://www.project-freya.eu/en.

11 https://graph.openaire.eu.

12 https://www.openaire.eu/openaire-and-eosc.

13 https://gitlab.eudat.eu/dmp/OpenAIRE-EUDAT-DMP-service-pilot/-/tree/master.

14 https://eudat.eu/eudat-cdi.

15 Wiki page: https://gitlab.eudat.eu/dmp/OpenAIRE-EUDAT-DMP-service-pilot/-/wikis/home.

16 http://catalogue.openaire.eu/service/openaire.ARGOS.

17 https://marketplace.eosc-portal.eu/services/argos?fromc=data-management.

18 The Horizon Europe template is also available in ARGOS.

19 Application Programming Interfaces.

20 https://orcid.org/.

21 https://providers.eosc-portal.eu/openapi.

22 Zenodo https://zenodo.org.

23 Digital Object Identifiers.

24 https://www.re3data.org/repository/r3d100010468.

25 RDA Hackathon on ma-DMPs: https://github.com/RDA-DMP-Common/hackathon-2020. A full report from ARGOS participation in the Hackathon will be published by the Data Science Journal (https://datascience.codata.org/).

26 https://www.rd-alliance.org/groups/active-data-management-plans.html.

27 https://vocabularies.coar-repositories.org/resource_types/c_ab20/.

28 https://support.datacite.org/docs/relationtype_for_citation.

29 https://support.datacite.org/docs/a-crosswalk-of-datacite-and-rda-mapping.

## Abbreviations

API: Application Programming Interface
DMP: Data Management Plan
DOI: Digital Object Identifier
EOSC: European Open Science Cloud
FAIR: Findability, Accessibility, Interoperability, Reusability
ma-DMPs: machine-actionable DMPs
OpenAIRE RG: OpenAIRE Research Graph
OSGs: Open Science Graphs
PID: Persistent Identifier
RDA: Research Data Alliance
RDM: Research Data Management
RG: Research Graph
RRI: Responsible Research & Innovation
SMEs: Small Medium Enterprises
URL: Uniform Resource Locator
